# Audiovisual Temporal Processing and Synchrony Perception in the Rat

**DOI:** 10.3389/fnbeh.2016.00246

**Published:** 2017-01-10

**Authors:** Ashley L. Schormans, Kaela E. Scott, Albert M. Q. Vo, Anna Tyker, Marei Typlt, Daniel Stolzberg, Brian L. Allman

**Affiliations:** ^1^Department of Anatomy and Cell Biology, Schulich School of Medicine and Dentistry, University of Western OntarioLondon, ON, Canada; ^2^Department of Physiology and Pharmacology, Schulich School of Medicine and Dentistry, University of Western OntarioLondon, ON, Canada

**Keywords:** audiovisual temporal synchrony, multisensory processing, animal model, lateral extrastriate visual cortex, electrophysiology, simultaneity judgment, temporal order judgment

## Abstract

Extensive research on humans has improved our understanding of how the brain integrates information from our different senses, and has begun to uncover the brain regions and large-scale neural activity that contributes to an observer’s ability to perceive the relative timing of auditory and visual stimuli. In the present study, we developed the first behavioral tasks to assess the perception of audiovisual temporal synchrony in rats. Modeled after the parameters used in human studies, separate groups of rats were trained to perform: (1) a simultaneity judgment task in which they reported whether audiovisual stimuli at various stimulus onset asynchronies (SOAs) were presented simultaneously or not; and (2) a temporal order judgment task in which they reported whether they perceived the auditory or visual stimulus to have been presented first. Furthermore, using *in vivo* electrophysiological recordings in the lateral extrastriate visual (V2L) cortex of anesthetized rats, we performed the first investigation of how neurons in the rat multisensory cortex integrate audiovisual stimuli presented at different SOAs. As predicted, rats (*n* = 7) trained to perform the simultaneity judgment task could accurately (~80%) identify synchronous vs. asynchronous (200 ms SOA) trials. Moreover, the rats judged trials at 10 ms SOA to be synchronous, whereas the majority (~70%) of trials at 100 ms SOA were perceived to be asynchronous. During the temporal order judgment task, rats (*n* = 7) perceived the synchronous audiovisual stimuli to be “visual first” for ~52% of the trials, and calculation of the smallest timing interval between the auditory and visual stimuli that could be detected in each rat (i.e., the *just noticeable difference* (JND)) ranged from 77 ms to 122 ms. Neurons in the rat V2L cortex were sensitive to the timing of audiovisual stimuli, such that spiking activity was greatest during trials when the visual stimulus preceded the auditory by 20–40 ms. Ultimately, given that our behavioral and electrophysiological results were consistent with studies conducted on human participants and previous recordings made in multisensory brain regions of different species, we suggest that the rat represents an effective model for studying audiovisual temporal synchrony at both the neuronal and perceptual level.

## Introduction

Within the mammalian brain, there exist functionally-specialized regions, such as the superior colliculus and higher-order cortical areas, which are populated by neurons capable of merging information from more than one sensory modality (e.g., hearing and vision; Meredith and Stein, 1986; Stein and Meredith, 1993; Barth et al., 1995; Schroeder and Foxe, 2002; Wallace et al., 2004; Ghazanfar and Schroeder, 2006; Allman et al., 2008a; Stein and Stanford, 2008; Allman et al., 2009; Xu et al., 2014). As shown in numerous species (for review see Stein and Meredith, 1993), the successful integration of auditory and visual information allows for behavioral improvements in the detection, localization and identification of the stimuli (Hershenson, 1962; Hirokawa et al., 2008; Gleiss and Kayser, 2012; Raposo et al., 2012; Siemann et al., 2014). For example, consistent with studies on humans (Calvert et al., 2000; Diederich and Colonius, 2004), rats are able to detect auditory and visual stimuli more quickly, when the cues are presented in combination compared to when either cue is presented alone (Hirokawa et al., 2008; Gleiss and Kayser, 2012). Related to this, the lateral extrastriate visual cortex (V2L) in rats has been identified as a cortical area mediating the improved reaction time to detect audiovisual stimuli, as pharmacological deactivation of this region results in a loss of multisensory facilitation (Hirokawa et al., 2008).

In addition to studying various detection and localization tasks, psychophysical testing in humans has investigated the perceived temporal synchrony of audiovisual stimuli. Classically, two perceptual tasks have been used to probe an observer’s ability to discern audiovisual temporal synchrony. In the temporal order judgment task, auditory and visual stimuli are presented at various stimulus onset asynchronies (SOAs), and the observers must judge the relative timing of the stimuli by stating which one came first or which came second (Spence et al., 2001; Stone et al., 2001; Zampini et al., 2005a; Vatakis et al., 2008b; Keetels and Vroomen, 2012; Stevenson et al., 2014; Binder, 2015). The simultaneity judgment task also includes the presentation of audiovisual stimuli at various SOAs; however, the observers now judge whether they perceived the stimuli to have been presented at the same moment in time or not (Spence et al., 2003; Zampini et al., 2003; Navarra et al., 2005; Vatakis et al., 2007, 2008b; Boenke et al., 2009; Keetels and Vroomen, 2012). Performance in these tasks can be used to calculate: (1) the observer’s *point of subjective simultaneity* (PSS), which describes the actual timing of the audiovisual stimuli when the observer is most unsure of the temporal order and; (2) the observer’s *just noticeable difference* (JND), which represents the smallest interval between the separately presented auditory and visual stimuli that can be detected reliably (Vatakis et al., 2008a; Vroomen and Stekelenburg, 2011; Keetels and Vroomen, 2012).

In recent years, numerous studies have contributed to our understanding of the factors that influence one’s perception of audiovisual temporal synchrony. For example, it is well established that the PSS and JND calculated from simultaneity- and temporal order judgment tasks can be significantly affected by a variety of experimental parameters, including the stimulus intensity (Boenke et al., 2009; Krueger Fister et al., 2016), stimulus duration (Boenke et al., 2009) and overall task conditions (Zampini et al., 2005a,b; Stevenson and Wallace, 2013) as well as one’s prior exposure to asynchronous stimuli (Fujisaki et al., 2004; Navarra et al., 2005; Vatakis et al., 2007, 2008b). At the same time, functional neuroimaging and electroencephalography studies have offered insight into the brain regions activated during audiovisual temporal synchrony tasks, as well as large-scale neural activity associated with the perceptual judgments (Bushara et al., 2001; Calvert and Thesen, 2004; Binder, 2015). Moreover, studies on various clinical populations (e.g., autism spectrum disorder (ASD), dyslexia and schizophrenia, for review see Wallace and Stevenson, 2014) have begun to identify the associated deficits that exist in audiovisual processing, as well as differences in brain activation during task performance. Despite the wealth of information gleaned from human studies, important issues remain to be fully resolved, such as the specific response properties of single neurons and their local circuits that contribute to the perception of temporal synchrony, as well as the cellular mechanisms, neuronal responses and network properties underlying the altered perception in clinical populations. Given the considerable advances that have been made in neuron-specific activation/silencing using opto- and chemogenetics as well as the emergence of transgenic rats that model aspects of human neuropsychiatric disorders, it is reasonable to suggest that such experimental tools may help to reveal the neural substrates underlying the perception of synchrony between the senses. At present, however, a considerable hurdle exists as we are not aware of any studies that have established behavioral tasks in rats that probe for the perception of audiovisual temporal synchrony.

In the present study, we endeavored to design and implement the first simultaneity- and temporal order judgment tasks in rats. Using the appetitive operant conditioning, we trained separate groups of adult rats to: (1) differentiate whether audiovisual stimuli at various SOAs were presented synchronously or not (i.e., simultaneity judgment task); or (2) determine the temporal order of audiovisual stimuli presented at various SOAs (i.e., temporal order judgment task). Ultimately, psychophysical curves were generated for both of the behavioral paradigms, and the PSS and JND were calculated for the temporal order judgment task. Furthermore, prior to commencing the design of the novel behavioral tasks, we first performed *in vivo* electrophysiological recordings in the V2L cortex of anesthetized rats to assess the response characteristics of the constituent neurons to audiovisual stimuli presented at SOAs which are commonly used in human psychophysical studies. Not only did we intend to use these data to help determine which audiovisual SOAs would be included in the novel behavioral tasks, but to our knowledge, this would be the first investigation of how neurons in the rat multisensory cortex integrate audiovisual stimuli presented at different temporal onsets.

## Materials and Methods

The present study included three experimental series that each used a separate group of adult male Sprague-Dawley rats (Charles River Laboratories, Inc., Wilmington, MA, USA). Rats were housed on a 12-h light-dark cycle with food and water *ad libitum*. All experimental procedures were approved by the University of Western Ontario Animal Care and Use Committee and were in accordance with the guidelines established by the Canadian Council of Animal Care.

### Experiment 1- Electrophysiological Recordings in the Lateral Extrastriate Visual Cortex (V2L)

#### Surgical Procedure

Adult male rats (*n* = 7; body mass: 420 ± 11.8 g) were anesthetized with ketamine (80 mg/kg; IP) and xylazine (5 mg/kg; IP) and fixed in a stereotaxic frame with blunt ear bars. The absence of a pedal withdrawal reflex was an indication of anesthetic depth, and supplemental doses of ketamine/xylazine were administered (IM) as needed. A midline incision was made in the skin of the scalp, and the tissue overlying the dorsal aspect of the skull was removed. A stainless steel screw was inserted in the left frontal bone to serve as an anchor for a headpost and as an electrical ground. A stereotaxic micromanipulator was used to measure 5.5 mm caudal to bregma, which represents an approximate rostral-caudal location of the V2L (Wallace et al., 2004; Hirokawa et al., 2008; Xu et al., 2014; Schormans et al., 2016), and a mark was made on the skull for later drilling. A craniotomy (2.5 mm × 3 mm; 4–7 mm caudal to Bregma) was performed in the left parietal bone to expose the cortex. Following the surgical procedure, the right ear bar was removed to provide free-field auditory stimulation of the right ear during electrophysiological recordings in the contralateral cortex. The rat was held in position throughout the entire duration of the experiment within the stereotaxic frame using the left ear bar and the headpost.

#### Electrophysiological Recordings

Extracellular electrophysiological recordings were performed in a dark, double-walled, sound-attenuating chamber (MDL 6060 ENV, WhisperRoom Inc., Knoxville, TN, USA). Neural signals were acquired using a 32-channel microelectrode array which consisted of a single shank with 32 recordings sites equally-spaced, spanning 1.5 mm in length (A1x32-10mm-50-177-A32; NeuroNexus Technologies, Ann Arbor, MI, USA). The microelectrode array was connected to a high-impedance headstage (NN32AC; Tucker-Davis Technologies, Alachua, FL, USA), and the electrophysiological signal was preamplified and digitized (two RA16SD Medusa preamplifiers; TDT), and sent to a RZ5 processing module via a fiber optic cable. The neuronal activity was detected online (digitally sampled at 25 kHz and bandpass filtered online at 300–3000 Hz) using a voltage threshold for spike detection of three standard deviations above the noise floor. The timing of the detected spikes and their associated waveforms were stored for offline analyses.

**Figure 1:**
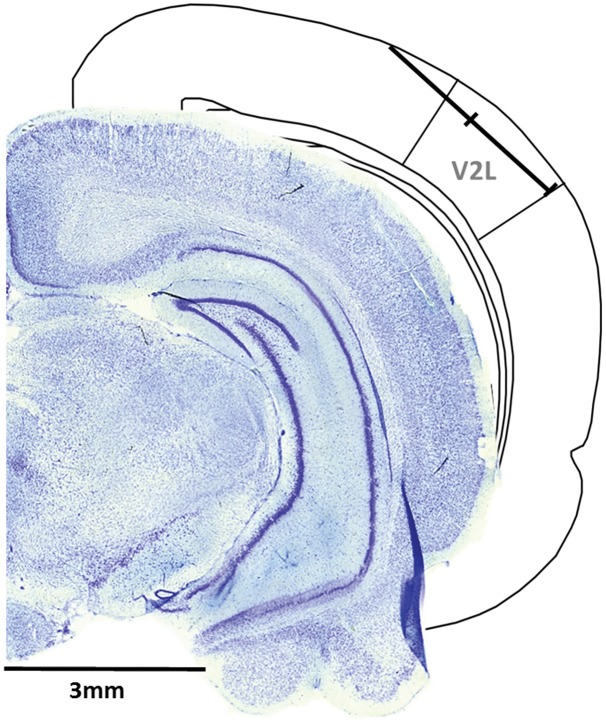
**Representative recording penetration in the lateral extrastriate visual (V2L) cortex**. A schematic of the recording location from the pial surface (1.75–3.25 mm) when the microelectrode array was advanced into the cortex at 5.5 mm caudal to Bregma using a dorsomedial-to-ventrolateral approach (40° angle). As shown in the coronal sections (figure adapted from Paxinos and Watson, 2007), the electrode array was positioned within the V2L cortex and was typically located within the supragranular and granular layers.

A single penetration was completed in each experiment, whereby the microelectrode array was inserted in the cortex through a small slit in the dura using a dorsomedial-to-ventrolateral approach (40° angle), with the array entering the cortex 5.5 mm caudal to bregma and approximately 1 mm medial to the temporal ridge of the skull. The array was inserted into the cortex using a stereotaxic micromanipulator (World Precision Instruments, Sarasota, FL, USA) at a 40° angle until all recording sites were within the cortex (depth of 1.5 mm) based on visual confirmation using a surgical microscope equipped with a high-resolution camera. Once the electrode sites were no longer visible, a hydraulic microdrive (FHC, Bowdoinham, ME, USA) was used to slowly advance the array into the cortex until the 32 recording sites spanned a distance of 1.75–3.25 mm from the initial entry in the cortex. At this location, the recording sites were located within the V2L, a multisensory region responsive to auditory and visual stimuli (Toldi et al., 1986; Barth et al., 1995; Wallace et al., 2004; Hirokawa et al., 2008; Xu et al., 2014; Schormans et al., 2016; Figure 1).

#### Audiovisual Stimulation Paradigms

Auditory stimuli consisted 50 ms noise bursts (1–32 kHz) from a speaker (MF1, TDT) positioned approximately 10 cm from the right pinna on a 30° angle from midline (i.e., speaker was positioned in the space contralateral to the electrode position). For each rat, the auditory stimulus (52.8 ± 1.5 dB sound pressure level, SPL) was presented at 30 dB above its threshold to a click (0.1 ms) stimulus (22.8 ± 1.5 dB SPL) as determined by an initial assessment of hearing sensitivity using our previously described auditory brainstem response paradigm (Schormans et al., 2016). Briefly, rats were anesthetized with ketamine and xylazine (IP) and subdermal electrodes were positioned at the vertex, over the right mastoid and on the back. The auditory stimulus consisted of a click (0.1 ms) which was presented at decreasing intensities from 90 dB to 10 dB SPL, in 10 dB SPL steps. Near threshold, the stimulus intensity was then presented at 5 dB SPL steps, and ABR threshold was determined using the criteria of JND of the averaged electrical activity within the 10 ms window (Popelar et al., 2008). The sound stimuli were calibrated using a ¼-inch microphone (2530; Larson Davis, Depew, NY, USA) and preamplifier (2221; Larson Davis) and custom Matlab software (The MathWorks, Natick, MA, USA). The visual stimulus consisted of a 50 ms flash of light (11 lux; centered on the eye) from a single LED (diameter: 0.8 cm) positioned adjacent the speaker. Based on pilot recordings and consistent with our earlier work (Schormans et al., 2016), a flash of light at 11 lux was chosen because it evoked a consistent, yet submaximal level of neuronal responsiveness, thereby allowing for the potential to observe enhanced multisensory responses during combined stimulus conditions (i.e., inverse effectiveness; Stein and Meredith, 1993).

Computer-triggered auditory and visual stimuli were presented alone or in combination using a TDT RZ6 processing module (100 kHz sampling rate) and custom Matlab software. Auditory and visual stimuli were presented alone in order to determine the sensory responsiveness of each of the multi-unit (MU) clusters sampled during the experiment. The combined audiovisual stimuli were presented at various SOAs in which the visual stimulus was presented either 80, 60, 40 or 20 ms before the auditory stimulus, at the same time as the auditory stimulus (0 ms onset), or 20, 40, 60 or 80 ms after the auditory stimulus. In addition to the auditory alone, visual alone and nine audiovisual conditions, the paradigm also included trials in which no stimulus was presented in order to collect spontaneous activity. Overall, the trial conditions were presented in a pseudorandomized order, separated by an inter-trial interval of 3–5 s, and each condition was presented 50 times.

#### Multi-unit Analysis and Multisensory Enhancement

**Figure 2:**
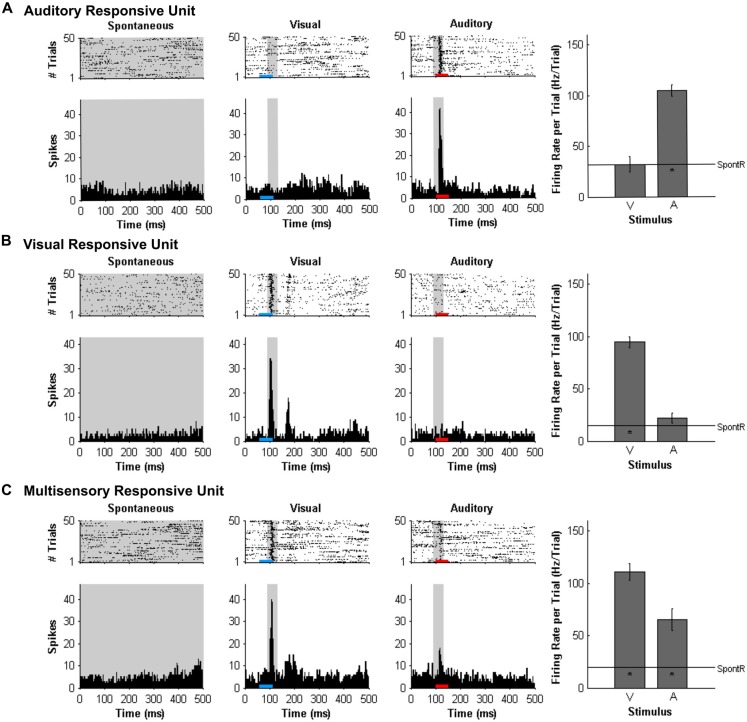
**Responses of multi-unit (MU) clusters to auditory, visual and combined audiovisual stimuli**. For a representative MU cluster, responses to no sensory stimulus (i.e., spontaneous activity; left panels), visual (50 ms LED flash at 11 lux, denoted by the blue horizontal bar; middle panels), and auditory (50 ms noise burst at 30 dB above click threshold, denoted by the red horizontal bar; right panels) are shown in the rasters (dot = spike; each row = 1 of 50 trials) and peri-stimulus time histograms (PSTH; 5 ms time bins). Spontaneous activity was determined in the no stimulus condition. For each MU cluster, firing rate in response to an auditory or visual stimulus was calculated within a 40 ms window (gray shading on rasters and PSTH; 90–130 ms) and average firing rate per trial ± SEM are shown in the bar graphs. In each bar graph, the “*” appearing below the horizontal line (spontaneous activity; SpontR) denotes whether a particular stimulus was effective at eliciting an overt response (see“Materials and Methods” Section for details). The MU clusters shown were classified as being responsive to auditory **(A)** visual **(B)** or both auditory and visual stimuli (i.e., multisensory; **C**).

At each of the 32 recording sites on the microelectrode array, MU activity was analyzed and the results described in terms of each MU cluster’s overall “sensory responsiveness” to the auditory and/or visual stimuli, as described previously (Schormans et al., 2016). For each MU cluster, custom Matlab scripts were used to generate rasters and PSTHs for each stimulus condition. To assess if a cluster was responsive to the auditory and/or visual stimuli, it had to demonstrate a significantly increased firing rate per trial compared to the spontaneous activity as determined with a paired *t*-test (*α* = 0.05; Allman and Meredith, 2007; Allman et al., 2008a; Schormans et al., 2016). Spontaneous activity was determined by first tallying the number of spikes within the 500-ms time window for each of the 50 trials, and then calculated by averaging the firing rate per trial over the 50 trials (SpontR; see Figure 2 for representative values). Figure 2 shows representative examples of MU clusters that were classified as being responsive to auditory (Figure 2A), visual (Figure 2B) or both auditory and visual stimuli (i.e., multisensory, Figure 2C).

**Figure 3:**
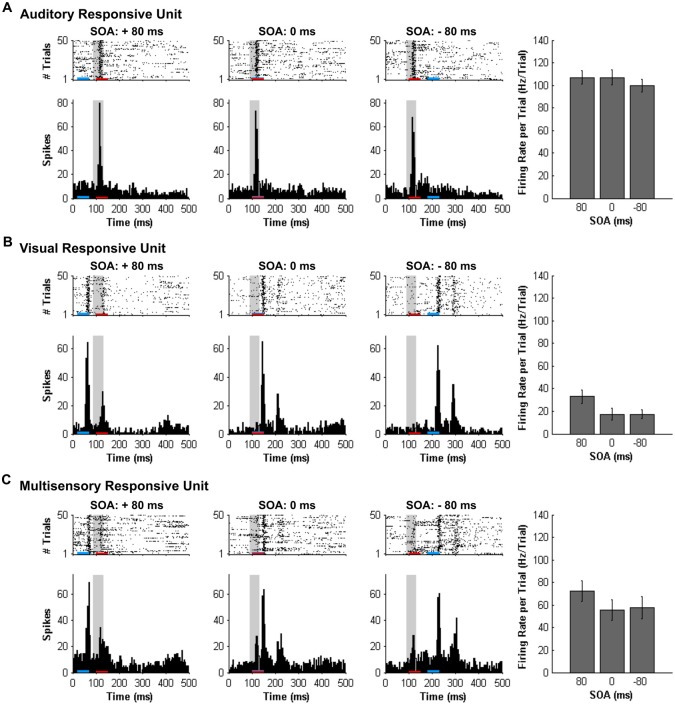
**Spiking activity of MU clusters at various audiovisual temporal onsets assessed using a set window analysis**. Rasters and PSTHs show the spiking activity of representative MU clusters (**(A)** auditory-responsive; **(B)** visual-responsive; **(C)** multisensory-responsive) to combined auditory (50 ms noise burst; denoted by the red bar) and visual (50 ms LED flash, denoted by the blue bar) at three different stimulus onset asynchronies (SOAs). At a SOA of +80 ms, the onset of the visual stimulus preceded the auditory stimulus by 80 ms (left rasters and PSTHs), whereas an SOA of −80 ms indicates that auditory stimulus preceded the visual stimulus by 80 ms (right rasters and PSTHs). A temporal difference of 0 ms represents the simultaneous presentation of the auditory and visual stimuli (middle rasters and PSTHs). For each responsive MU cluster, the mean firing rate per trial ± SEM (shown in the bar graphs) was calculated based on a 40-ms window fixed in time (i.e., set window). The set window analysis captured the majority of the spiking activity of auditory- and multisensory-responsive MU clusters; however, because the onset of the visual stimulus moved in time, the set window failed to consistently capture the maximal responsiveness of the visual MU cluster across all SOAs (note the low firing rates in bar graphs).

**Figure 4:**
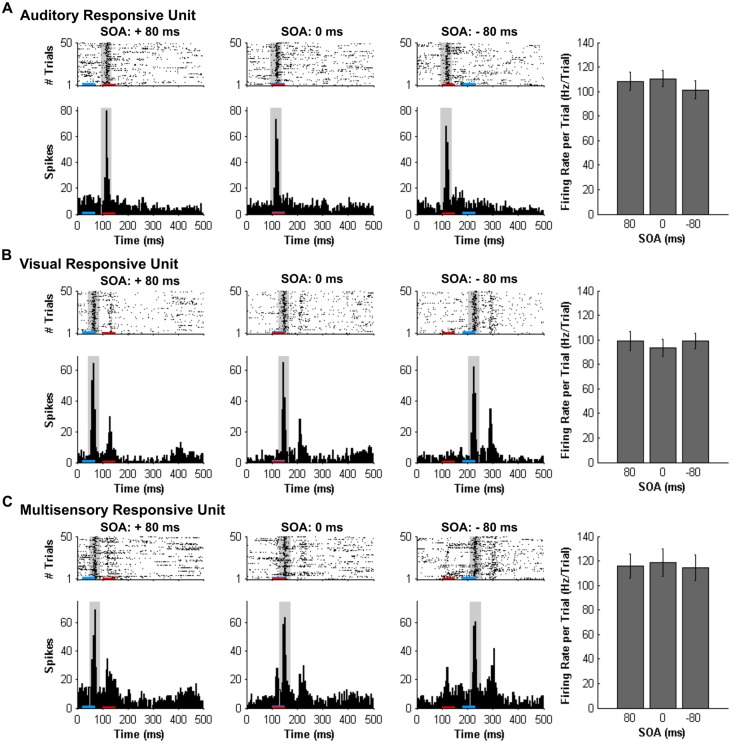
**Spiking activity of MU clusters at various audiovisual temporal onsets assessed using an analysis window centered on the peak firing rate**. Rasters and PSTHs show the spiking activity of the same representative MU clusters shown in Figure 3. **(A)** auditory-responsive; **(B)** visual-responsive; **(C)** multisensory-responsive) to combined auditory (50 ms noise burst; denoted by the red bar) and visual (50 ms LED flash, denoted by the blue bar) at three different stimulus onset asynchronies (SOAs). At a SOA of +80 ms, the onset of the visual stimulus preceded the auditory stimulus by 80 ms (left rasters and PSTHs), whereas an SOA of −80 ms indicates that auditory stimulus preceded the visual stimulus by 80 ms (right rasters and PSTHs). A temporal difference of 0 ms represents the simultaneous presentation of the auditory and visual stimuli (middle rasters and PSTHs). For each responsive MU cluster, the mean firing rate per trial ± SEM (shown in the bar graphs) was calculated based on a 40-ms window (gray shading on rasters and PSTH) centered on the peak firing rate within the sampling window. For example, the location of the 40 ms peak-centered window for the visual-responsive MU cluster was different at each SOA, given that the onset of the visual stimulus was moved in time with respect to the static auditory stimulus (presented 100 ms from the beginning of the trial). Consequently, the mean firing rate per trial ± SEM (seen in the bar graphs) of the visual-responsive MU cluster **(B)** was similar across SOAs, consistent with the auditory-responsive MU cluster **(A)**.

Consistent with prior studies (King and Palmer, 1985; Lippert et al., 2013), all responsive MU clusters underwent analyses to determine its mean firing rate for each of the stimuli conditions using two methods: (1) a firing rate calculation based on latency of auditory responses (i.e., firing rate calculated from 90 ms to 130 ms from trial onset; “set window”); and (2) a firing rate calculation window based on the latency of the peak firing rate irrespective of stimulus (i.e., firing rate calculated from 40 ms window centered around the peak firing rate within the overall 500 ms sampling time; “peak-centered window”). Figures 3, 4 show representative calculation windows (i.e., gray shading on the rasters and PSTHs) for the mean firing rate derived from the “set window” and “peak-centered window” conditions, respectively. Table 1 shows the average start time for the 40 ms peak-centered window across all audiovisual SOAs presented. A “set window” of 90–130 ms was selected based on previous recordings within the V2L cortex, as this timing window captured the vast majority of auditory and visual responses of single- and MU clusters (Schormans et al., 2016). Overall, a series of calculations were performed to generate an average response profile across all animals associated with the set window and peak-centered window analyses. Prior to the group calculations, the following steps were performed on MU clusters collected from each rat. First, using the set window for example, the firing rate per trial of a given auditory-responsive MU cluster (e.g., the one depicted in Figure 3A) was divided by the firing rate per trial of the most effective unimodal stimulus condition (e.g., Figure 2A, auditory response) to calculate the percent change in firing rate at each of the audiovisual SOAs (i.e., 0, ±20, ±40, ±60 and ±80 ms). This calculation was used to describe the degree of change due to the timing of the audiovisual stimuli, and presented as the level of multisensory enhancement. Next, for a given rat, all of its auditory-responsive MU clusters were averaged at each of the SOAs for both the mean firing rate and the level of multisensory enhancement using the set window. Ultimately, the aforementioned series of calculations were performed on all of the auditory-, visual- and multisensory-responsive MU clusters sampled from each rat using both the set window and peak-centered window analyses. Finally, an average was derived from all seven rats at each of the audiovisual SOAs for the mean firing rate and multisensory enhancement.

**Table 1:** **Start time of the 40 ms peak-centered window across stimulus onset asynchronies (SOAs) for auditory, visual and multisensory multi-unit clusters**.

	Auditory		Visual		Multisensory	
SOA	Mean (ms)	SEM	Mean (ms)	SEM	Mean (ms)	SEM
**+80 ms**	95.3	0.2	43.9	0.9	74.1	4.4
**+60 ms**	95.7	0.3	66.5	1.3	83.8	2.6
**+40 ms**	95.1	0.1	84.4	0.8	91.9	0.8
**+20 ms**	95.5	0.2	102.8	0.5	100.7	0.8
**0 ms**	95.4	0.2	123.8	1.1	109.9	2.7
**−20 ms**	97.4	1.9	141.6	1.4	117.8	4.1
**−40 ms**	95.6	0.3	162.3	0.8	130.0	6.1
**−60 ms**	95.7	0.2	181.9	1.4	136.3	7.7
**−80 ms**	95.5	0.2	198.7	2.2	139.0	9.4

#### Histology

Following the completion of the electrophysiological recordings, the rat was injected with sodium pentobarbital (100 mg/kg; IP) in preparation for a transcranial perfusion with 0.1 M phosphate buffer (PB), followed by 4% paraformaldehyde. The brain was then removed and post-fixed in paraformaldehyde for 12 h, followed by storage in 30% sucrose. Coronal sections (40 μm) were cut using a freezing microtome (HM 430/34; Thermo Scientific, Waltham, MA, USA). After staining with thionin, the coronal sections were imaged with an Axio Vert A1 inverted microscope (Carl Zeiss Microscopy GmbH, Jena, Germany). ZEN imaging software was used to reconstruct the location of each recording penetration (see Figure 1 for representative image).

### Experiment 2- Simultaneity Judgment Task

A separate group of adult male rats (*n* = 7; training began at 70 days old; body mass: 286 ± 4.4 g) were trained 6 days per week using a two-alternative forced-choice operant conditioning paradigm to differentiate between trials when a visual stimulus was presented simultaneously with an auditory stimulus (0 ms onset; synchronous), or when the visual stimulus preceded the auditory stimulus by 200 ms (i.e., asynchronous). As described in detail below, once the rats were proficient at the training task, occasional testing days occurred in which novel SOAs were also added to the paradigm whereby the visual stimulus preceded the auditory stimulus by 0, 10, 40, 100 or 200 ms. These testing days took place when the rats were between 6 and 11 months of age (body mass at the last day of testing: 449 ± 16.3 g), and allowed for the determination of each rat’s judgment of simultaneity.

#### Behavioral Apparatus and Sensory Stimuli

Behavioral training was performed using a standard modular test chamber (ENV-008CT; Med Associates Inc., St. Albans, VT, USA), which was housed in a sound-attenuating box (29″ W by 23.5″ H by 23.5″ D, Med Associates Inc., St. Albans, VT, USA). The behavioral chamber was illuminated by a house light located on the back wall, whereas the front wall was equipped with a center nose poke, a left feeder trough and a right feeder trough; each fitted with an infrared (IR) detector. Stimulus delivery, nose-poke responses and positive/negative reinforcement were controlled and monitored using custom Matlab behavioral protocols running in Matlab (EPsych Toolbox^1^) which was interfaced with real-time processing hardware (RZ6, TDT). The visual stimulus consisted of a light flash (27 lux; 50 ms duration) from an LED (ENV-229M; Med Associates Inc.) located above the center nose poke. The intensity of the visual stimulus (as determined by a LED light meter; Model LT45, Extech Instruments, Nashua, NH, USA) was constrained by the hardware associated with the operant conditioning chamber (Med Associates Inc.). The auditory stimulus was a noise burst (1–32 kHz; 75 dB SPL; 50 ms duration) from a speaker (FT28D, Fostex, Tokyo, Japan) mounted on the ceiling of the behavioral chamber near the front wall. Pilot studies revealed that the rats had difficulty learning either paradigm to a performance criterion of 75% when a lower sound level (i.e., 60 dB SPL) was used; findings which are consistent with studies demonstrating improved audiovisual temporal discrimination with increasing sound intensities (Boenke et al., 2009; Krueger Fister et al., 2016). The intensity of the auditory stimulus was calibrated with custom Matlab software using a 1/4″ microphone (2530, Larson Davis) and preamplifier (2221; Larson Davis). The duration of the stimuli (i.e., 50 ms) was not varied in order to be consistent with electrophysiological recordings.

#### Behavioral Training

Prior to commencing behavioral training, the rats were weighed daily and maintained on a food restricted diet until they reached 85% of their free feeding body mass. Initially, the rats were habituated to the behavioral chamber for 30 min/day. During these habituation sessions, spontaneous nose pokes into the center port (detected by the IR beam) resulted in: (1) the immediate presentation of an audiovisual stimulus combination that was either synchronous (i.e., 0 ms onset) or asynchronous (i.e., visual stimulus 400 ms prior to auditory stimulus); and (2) the delivery of a 45 mg food pellet (Bio-Serv, Frenchtown, NJ, USA) to the associated feeder trough (i.e., synchronous = left feeder trough; asynchronous = right feeder trough). Furthermore, if the rat went to the correct feeder trough following the stimuli presentation (as monitored with the IR detector), a second pellet was delivered so as to help the rat associate a given feeder trough with a particular audiovisual SOA.

**Figure 5:**
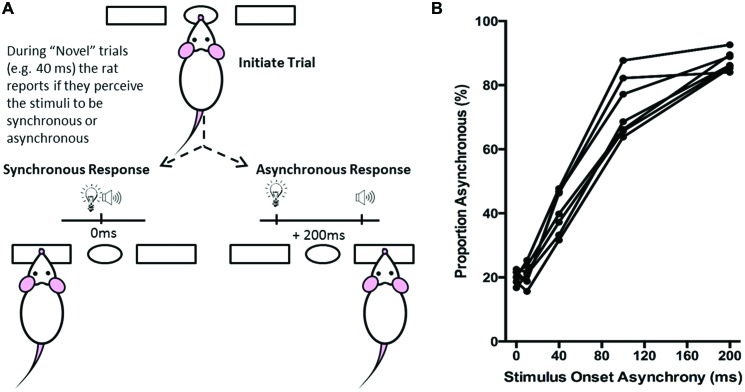
**Overview of simultaneity judgment task. (A)** The simultaneity judgment task consisted of the rat initiating a trial by poking its nose into the center port, and holding for up 2 s. In response to the presentation of an audiovisual stimulus, the rat was trained to respond to the left feeder trough for the synchronous (0 ms SOA) trials and to the right feeder trough for the asynchronous (200 ms SOA) trials. On testing days, upon presentation of novel SOAs (0, 40 and 100 ms), the rat reported whether it judged the audiovisual stimuli to have been presented synchronously or asynchronously. **(B)** The behavioral performance of individual rats was plotted as the proportion of responses that the rat judged as asynchronous (i.e., right feeder trough). Each data point represents the average of five psychophysical testing sessions for an individual rat (*n* = 7).

Once the rats were able to frequently nose poke in the center port (typically within 3 days), the initial pellet reinforcement was removed, and now the pellet delivery was contingent on the rat poking its nose in the correct feeder trough in response to the given audiovisual SOA. At this stage, the audiovisual asynchronous stimuli onset remained at 400 ms. During each 30-min daily training session, correct feeder trough responses were reinforced with a food pellet, whereas incorrect responses resulted in the house light turning off for 15 s, during which time the rat was unable to initiate a new trial (Figure 5A). Throughout the behavioral training, the amount of food provided in each rat’s home cage was adjusted so that its body mass increased with age while still providing enough motivation for it to perform ~200 trials in a session (Stolzberg et al., 2013).

Rats remained on the 0 ms vs. 400 ms SOA protocol until they correctly identified the synchronous and asynchronous audiovisual combinations with >75% accuracy. Upon achieving this performance criterion for three consecutive days, the asynchronous SOA was reduced to 300 ms. Training continued in sessions of 30 min/day or to the completion of 200 trials until a criterion of 75% correct was reached for both synchronous and asynchronous stimuli on five consecutive days. During the final training stage of the simultaneity judgment task, the asynchronous stimuli onset was reduced to 200 ms. As described below, after the rat had achieved >80% accuracy for five consecutive days on the final training stage (i.e., 0 ms vs. 200 ms SOA), “testing” days were performed approximately once a week.

#### Behavioral Testing and Analysis

To determine each rat’s perception of simultaneity (i.e., whether it judged a given audiovisual stimuli combination as being presented synchronously or asynchronously), novel SOAs were introduced. On average, rats underwent testing once a week, in which five SOAs were randomly presented (i.e., the visual stimulus preceded the auditory stimulus by 0, 10, 40, 100 or 200 ms; see Figure 5B), whereas the other days of the week remained as training sessions (i.e., only 0 ms vs. 200 ms SOA). On testing days, the familiar 0 ms and 200 ms SOAs continued to be reinforced with food pellets for correct responses and 15-s timeouts for incorrect responses; however, the novel temporal onsets (i.e., 10, 40, and 100 ms SOA) were reinforced regardless of whether a correct or incorrect response was made. For the majority (70%) of trials on test days, the rats were presented with the 0 ms or 200 ms SOAs, whereas the remaining 30% of the trials were divided equally between the 10, 40 and 100 ms SOAs. Pilot testing revealed that this trial breakdown helped to prevent the rats from developing a side bias to the novel SOAs.

Ultimately, the simultaneity judgment task was designed such that if the rat perceived the audiovisual stimuli to have been presented synchronously, it would respond by nose-poking the left feeder trough, whereas if it perceived the audiovisual stimuli to have presented asynchronously, it would respond by nose-poking the right feeder trough (Figure 5A). Each rat completed a total of five test sessions over a 2 month period, from which its performance on each of the SOAs (0, 10, 40, 100 and 200 ms) was reported as the proportion of trials that were judged as asynchronous (i.e., % right feeder trough responses; Figure 5B). Test days were repeated if the performance on the training SOAs (i.e., 0 ms and 200 ms) fell below the criterion of 70% correct. Finally, to determine each rat’s baseline performance on the simultaneity judgment task, the results from the five successful test days were averaged for the various SOAs to create a psychophysical profile (Figure 5B).

### Experiment 3- Temporal Order Judgment Task

Using the same behavioral apparatus and sensory stimuli described in Experiment 2, a separate group of adult male rats (*n* = 7; training began at 70 days old; body mass: 310 ± 4.9 g) were trained 6 days per week using a two-alternative forced-choice operant conditioning paradigm to differentiate the temporal order of auditory and visual stimuli (i.e., which stimulus modality was presented first when separated by 200 ms). As outlined in the following sections, once the rats were proficient at the training task, occasional testing days occurred in which novel SOAs (0, ±40 and ±100 ms) were also added to the paradigm. Ultimately, the testing days, which took place when the rats were between six and eight months of age (body mass at the last day of testing: 422 ± 11.6 g), allowed for the determination of each rat’s perception of audiovisual temporal order.

#### Behavioral Training

**Figure 6:**
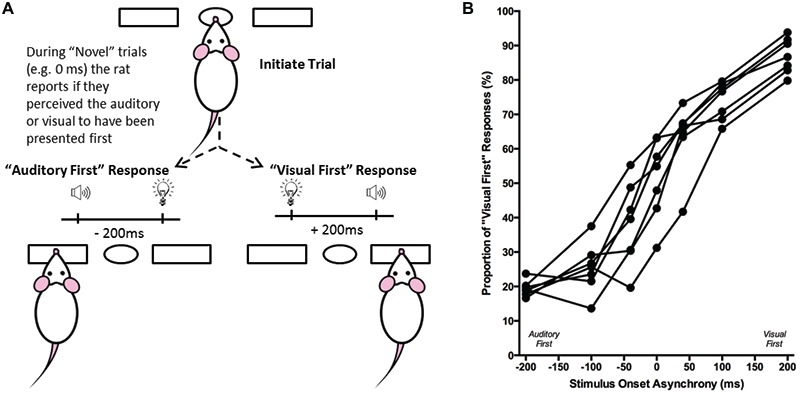
**Overview of temporal order judgment task**. **(A)** The temporal order judgment task consisted of the rat initiating a trial by poking its nose into the center port, and holding for up 2 s. In response to the presentation of an audiovisual stimulus, the rat was trained to respond to the left feeder trough on trials when the auditory stimulus preceded the visual (−200 ms SOA), and to the right feeder trough when the visual stimulus was presented first (+200 ms SOA). On testing days, when the rat was presented novel SOAs (0, ±40 and ±100 ms), it reported whether it judged the audiovisual stimuli to have been “auditory first” or “visual first”. **(B)** The behavioral performance of individual rats was plotted as the proportion of responses that the rat judged as “visual first” (i.e., right feeder trough). Each data point represents the average of five psychophysical testing sessions for an individual rat (*n* = 7).

Several aspects of the behavioral training were consistent with those described above in Experiment 2, such as the food deprivation, habituation, general nose-poking procedures, session duration (30 min/day or ~200 trials), frequency of training (6 days per week), positive/negative reinforcement, as well as an incremental progression through the various training stages. Importantly, in contrast to the simultaneity judgment task, rats in Experiment 3 received a food pellet for nose-poking the left feeder trough when the auditory stimulus preceded the visual stimulus by 400 ms, and for nose-poking the right feeder trough when the visual stimulus was presented 400 ms before the auditory stimulus (Figure 6A). Once the rats reached the performance criterion of 75% correct for three consecutive days at a temporal onset of ±400 ms, the SOAs were reduced to ±300 ms. Moreover, when the rat scored >75% correct for five consecutive days, the SOAs were reduced to ±200 ms for the final training stage of the temporal order judgment task. As described below, behavioral testing days were performed approximately once a week after the rats had achieved >80% accuracy on five consecutive training days.

#### Behavioral Testing and Analysis

On testing days, novel SOAs were introduced so as to determine each rat’s perception of the temporal order of the auditory and visual stimuli. On average, rats underwent testing days once a week, in which seven SOAs were randomly delivered (i.e., 0, ±40, ±100 and ±200 ms; see Figure 6B), with the other days of the week remaining as training days (i.e., only the ±200 ms). On testing days, food pellets were delivered following the novel SOAs (0, ±40 and ±100 ms) regardless of whether a correct or incorrect response was made. In contrast, the audiovisual stimuli conditions familiar to the rat through training (i.e., ±200 ms) continued to be reinforced with food pellets for correct responses and 15-s timeouts for incorrect responses. To help avoid the potential development of a side bias during testing days, the training stimuli were presented for the majority (70%) of the trials, with the other 30% of trials divided between the novel SOAs.

Performance at each of the SOAs was measured as the proportion of trials in which the rat responded on the right feeder trough (i.e., visual first; Figure 6B). Test days were repeated if the trained stimuli (i.e., ±200 ms) did not reach the criterion of 70% correct. Ultimately, the results at the seven SOAs (0, ±40, ±100 and ±200 ms) were averaged across the five successful test days to create a psychophysical profile of each rat’s audiovisual temporal order judgment (Figure 6B). Moreover, best-fitting straight lines were plotted between each of the neighboring SOAs tested (e.g., −200 ms to −100 ms; −100 ms to −40 ms; etc.), and the associated slopes and intercept values were tabulated. From these values, the PSS was calculated by determining the SOA at which 50% of the responses were “visual first” (Vatakis et al., 2007). Similar to the PSS, the JND was calculated by taking the difference between the SOAs at which 25% and 75% of the responses were considered “visual-first” and then dividing by two (Vroomen and Stekelenburg, 2011). For each rat, PSS and JND were determined on the each of the five testing days, and the average PSS and JND values were calculated.

### Statistics and Data Presentation

Overall, the statistical analyses performed in the present study included one-way repeated-measures analysis of variance (ANOVA), and paired samples *t*-tests, depending on the comparison of interest (see “Results” Section for the details of each specific comparison). If Mauchly’s test of sphericity was violated within the repeated-measures ANOVA, the Greenhouse-Geisser correction was used. The level of statistical significance was set at *α* = 0.05. When appropriate, Bonferroni *post hoc* corrections were used to account for potential “family-wise” error (Armstrong, 2014). SPSS software (version 20, IBM Corporation, Armonk, NY, USA) was used for the statistical analyses. Matlab and GraphPad Prism (GraphPad Software Inc., La Jolla, CA, USA) were used to plot the results. Data are presented as the mean values ± standard error of the mean (SEM).

## Results

### Experiment 1- Electrophysiological Recordings in the Lateral Extrastriate Visual Cortex (V2L)

All rats (*n* = 7) included in this experimental series underwent the same electrophysiology recording procedure, which consisted of a single penetration of the 32-channel microelectrode array into the V2L. In total, 224 waveform clusters were sampled, with 221 (98.7%) of these MU clusters being classified as responsive to at least one sensory modality. Of the MU clusters that were responsive to sensory stimuli, 97 (43.9%) were overtly responsive to only the visual stimulus, 90 (40.7%) were overtly responsive to only the auditory stimulus, and 34 (15.4%) were overtly responsive to both the auditory and visual stimuli (i.e., multisensory MU clusters). As described in the “Materials and Methods” Section, the mean firing rate and level of multisensory enhancement of each MU cluster were determined at the various audiovisual SOAs (i.e., 0, ±20, ±40, ±60 and ±80 ms). These calculations were performed when the analysis window was either fixed at a given time interval (i.e., set window: from 90 to 130 ms from the start of the trial) or when it was shifted according to the peak firing rate (i.e., peak-centered window).

#### Mean Firing Rate and Multisensory Enhancement Calculated from a Set Window

**Figure 7:**
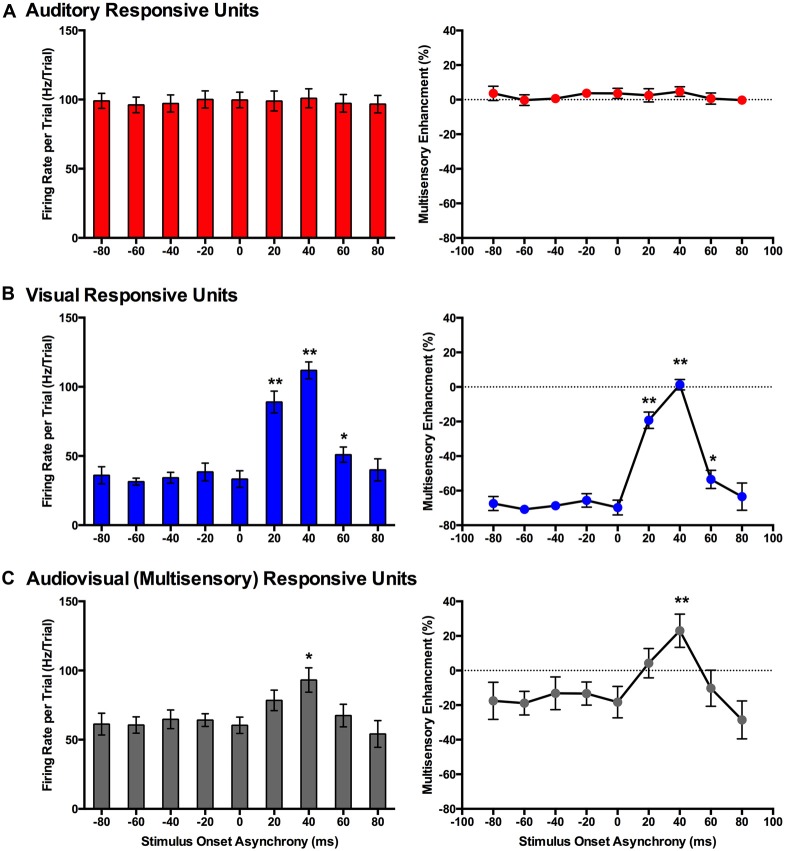
**Use of a set window analysis to compare the spiking activity of MU clusters evoked from audiovisual stimuli presented at various temporal onsets**. For MU clusters that were responsive to visual, auditory and both auditory and visual stimuli (i.e., multisensory), the group mean firing rate (left panels) and the level of multisensory enhancement (right panels) were determined based on a set window of analysis that was fixed at 90–130 ms from the start of the trial (see Figure 3 for representative rasters and PSTHs). Auditory-responsive MU clusters **(A)** showed no effect of stimulus onset asynchrony (SOA), whereas clusters that exclusively responded to visual stimuli **(B)** showed an increase in mean firing rate and multisensory enhancement at a SOA of +20 (**p < 0.0125), +40 (**p < 0.0125) and +60 ms (*p < 0.05) when compared to the synchronous presentation of stimuli (i.e., 0 ms SOA). **(C)** Multisensory responsive clusters showed an increase in mean firing rate (*p < 0.05) and multisensory enhancement (**p < 0.0125) at a SOA of +40 ms when compared to a SOA of 0 ms. Results are displayed as mean ± SEM, *n* = 7. Statistical comparisons are based on a repeated-measures ANOVA and Bonferroni corrected *post hoc* tests in which the significant *p*-value was adjusted to **p < 0.0125 to account for the multiple comparisons.

As shown in Figure 7A, separate one-way repeated-measures ANOVAs revealed that both the mean firing rates (*F*_(3.7,22.1)_ = 0.693, *p* = 0.593) and level of multisensory enhancement (*F*_(3.0,18.1)_ = 0.666, *p* = 0.585) of auditory-responsive MU clusters were not significantly affected by the various SOAs. This finding was not surprising given that the timing of the auditory stimulus did not vary during the SOA protocol; the onset of the visual stimulus shifted around the static auditory stimulus. Thus, because the spiking activity of the auditory-responsive MU clusters was consistently captured in the set window (see gray bars in Figure 3A) and these neurons, by definition, did not show overt responsiveness to the visual stimulus, it was expected that the mean firing rates and level of multisensory enhancement would be largely unaffected by the varying SOAs.

In contrast to the auditory-responsive MU clusters, the spiking profiles of neurons that responded exclusively to the visual stimulus were significantly affected by the set window analysis, as the fixed window often failed to capture the visually-evoked activity (see Figure 3B; gray set window does not overlap maximum spiking response). Thus, it was not surprising that a one-way repeated-measures ANOVA revealed a significant effect of SOA on the mean firing rate (*F*_(3.1,18.6)_ = 64.186, *p* < 0.001), and Bonferroni corrected *post hoc* analyses revealed that the mean firing rate was significantly greater at +20, +40 and +60 ms SOA compared to the synchronous presentation of the audiovisual stimuli (i.e., 0 ms onset). Similarly, the level of multisensory enhancement was significantly greater at the +20, +40 and +60 ms SOA than when the audiovisual stimuli were presented synchronously, as determined by a one-way repeated measures ANOVA (*F*_(3.2,18.9)_ = 57.049, *p* < 0.001) and Bonferroni corrected *post hoc* testing (*p* < 0.0125). Notice, however, that the level of multisensory enhancement in the visually-responsive MU clusters was well below 0% for the majority of the SOAs; again, an expected result due to the set window of analysis failing to capture the spiking evoked by the visual stimulus that moved in time.

Based on the set window analysis (Figure 7C), separate one-way repeated-measures ANOVAs revealed a significant effect of SOA on the mean firing rate (*F*_(2.3,9.2)_ = 6.201, *p* < 0.02) and level of multisensory enhancement (*F*_(2.2,8.7)_ = 6.313, *p* < 0.02) observed in multisensory-responsive MU clusters. Furthermore, *post hoc* analyses found a increase in mean firing rate (*p* < 0.05) and multisensory enhancement (*p* < 0.01) at +40 ms SOA compared to when the audiovisual stimuli were presented synchronously (0 ms SOA; Figure 7C).

#### Mean Firing Rate and Multisensory Enhancement Calculated from a Peak-Centered Window

**Figure 8:**
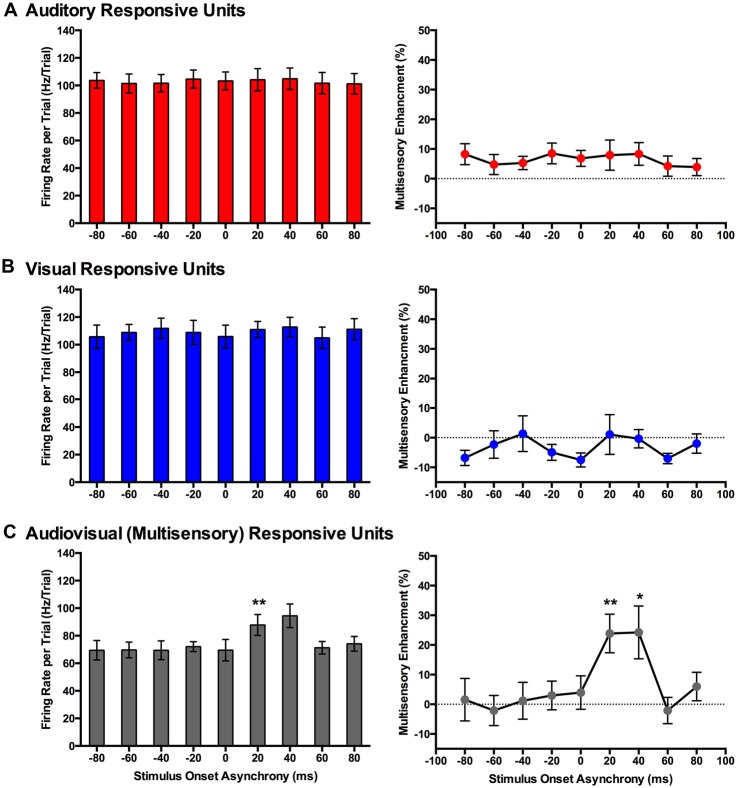
**Use of an analysis window centered on the peak firing rate to compare spiking activity of MU clusters evoked from audiovisual stimuli presented at various temporal onsets**. For MU clusters that were responsive to visual, auditory and both auditory and visual stimuli (i.e., multisensory), the group mean firing rate (left panels) and the level of multisensory enhancement (right panels) were determined based on a the latency of the peak firing rate within the sampling window for each MU cluster. For MU clusters that exclusively responded to auditory **(A)** or visual **(B)** stimuli, there was no effect of SOA on either the mean firing rate or level of multisensory enhancement. **(C)** Multisensory-responsive MU clusters showed an increase in mean firing rate and multisensory enhancement at a SOA of +20 ms when compared to 0 ms (**p < 0.0125). Moreover, at an SOA of +40 ms, an increase in multisensory enhancement was observed (*p < 0.05). Results are displayed as mean ± SEM, *n* = 7. Statistical comparisons are based on a repeated-measures ANOVA and Bonferroni corrected *post hoc* tests in which the significant *p*-value was adjusted to **p < 0.0125 to account for the multiple comparisons.

Similar to the results found using a set window, separate one-way repeated measures ANOVAs revealed that both the mean firing rates (*F*_(3.5,20.9)_ = 0.616, *p* = 0.635) and level of multisensory enhancement (*F*_(2.9,17.4)_ = 0.707, *p* = 0.556) of auditory-responsive MU clusters did not significantly differ across the various SOAs when a peak-centered window of analysis was used (Figure 8A). As shown in Figure 4B compared to Figure 3B, a peak-centered window of analysis better captured the stimulus-evoked spiking activity of visually-responsive MU clusters than a set window. Consequently, in contrast to Figure 7B (set window), separate one-way repeated-measures ANOVAs did not report a significant effect of SOA on the mean firing rates (*F*_(2.0,12.2)_ = 1.177, *p* = 0.342) or level of multisensory enhancement (*F*_(1.7,9.9)_ = 1.853, *p* = 0.208) observed in visually-responsive MU clusters (Figure 8B). The lack of effect of SOA on auditory- or visual-responsive MU clusters was not surprising given that these neurons had only shown overt spiking activity in response to a single modality (see Figures 2A,B for representative examples).

Consistent with the findings using the set window of analysis, multisensory-responsive MU clusters showed a sensitivity to SOAs when the visual stimulus preceded the auditory stimulus. Separate one-way repeated-measures ANOVAs revealed a significant effect of SOA on the mean firing rates (*F*_(1.9,7.4)_ = 5.466, *p* < 0.05) and level of multisensory enhancement (*F*_(2.4,9.4)_ = 7.902, *p* < 0.01) of multisensory-responsive MU clusters. Furthermore, *post hoc* analyses found an increase in mean firing rate and multisensory enhancement at +20 ms and +40 ms SOAs compared to when the audiovisual stimuli were presented synchronously (0 ms SOA; Figure 8C). Based on these electrophysiological results, we aimed to design novel behavioral paradigms that would assess rats’ ability to judge the simultaneity (Experiment 2) and temporal order (Experiment 3) of audiovisual stimuli specifically when the visual stimulus was presented 40 ms prior to the auditory stimulus.

### Experiment 2- Simultaneity Judgment Task

**Figure 9:**
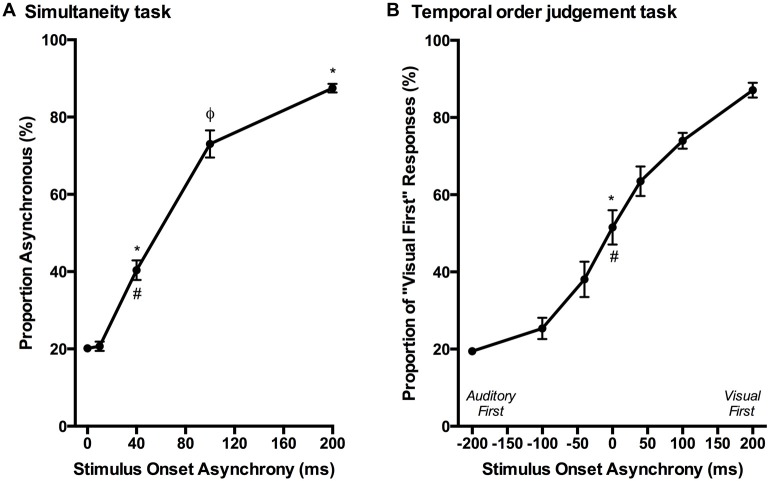
**Psychophysical profiles for the simultaneity judgment task and temporal order judgment task. (A)** Rats performing the simultaneity judgment task reported whether they perceived the audiovisual stimuli at various SOAs to have been presented synchronously or asynchronously (i.e., visual stimulus before the auditory). A significant difference in performance was observed between the simultaneous presentation of audiovisual stimuli (i.e., 0 ms) and 40 ms SOA (*p < 0.001) as well as 200 ms SOA (*p < 0.001); however, no significant difference was found between 0 ms and 10 ms SOA (*p* = 0.654). Additional statistical comparisons demonstrated that the performance at 40 ms SOA was significantly different from 200 ms SOA (^#^*p* < 0.001) and 100 ms SOA (^φ^*p* < 0.001). **(B)** The temporal order judgment task required rats to report whether an auditory or visual stimulus was perceived to have been presented first in the audiovisual pair. When stimuli were presented synchronously (0 ms SOA), rats on average perceived the stimuli to be “visual first” 52% of the time, which was significantly different than their performance at −200 ms SOA (*p < 0.001) and +200 ms SOA (^#^*p* < 0.001). Results are displayed as mean ± SEM, *n* = 7.

Over a series of stages, rats were trained using a two alternative forced choice paradigm to differentiate between audiovisual stimuli that were presented synchronously (0 ms SOA) and when the onset of the visual stimulus preceded the auditory stimulus by 200 ms. On average, training took place over 131 ± 7 days before they were able to undergo the testing procedures. Once the rats had become proficient at the training paradigm, five testing days were performed over the next two to three months in which novel audiovisual temporal onsets (10, 40 and 100 ms SOA) were presented. At the 40 ms SOA, rats perceived the stimuli to be asynchronous on 40 ± 2.6% of the trials (Figure 9A). A one-way repeated measures ANOVA revealed a significant main effect of SOA on the proportion of trials judged as asynchronous (*F*_(4,24)_ = 366.024, *p* < 0.001), and Bonferroni corrected *post hoc* analyses found that the performance during the 40 ms SOA was significantly different from all of the other SOAs tested (*p* < 0.001; Figure 9A). Moreover, the relatively short 10 ms SOA was also tested so that the face validity of the paradigm could be assessed, as human subjects judge audiovisual stimuli presented at 20 ms SOA to be synchronous (Zampini et al., 2005a). Consistent with these findings, the performance of the rats at the 10 ms SOA did not differ (*p* = 0.654) from that of the synchronous trials. Collectively, these results provide a psychophysical profile of simultaneity judgment in rats.

### Experiment 3- Temporal Order Judgment Task

Although the results of Experiment 2 were largely consistent with previous studies on humans, it is important to note that the tasks differed between species; unlike human subjects, the rats were only required to judge the simultaneity of the audiovisual stimuli when the visual stimulus preceded the auditory stimulus, and not vice-versa. Thus, in Experiment 3, we trained a separate group of rats to perform a temporal order judgment task in which they learned to differentiate between trials when the auditory stimulus either preceded or followed the visual stimulus by 200 ms. On average, the rats took 97 ± 7 days to reach the performance criterion required to advance to the five testing days, at which time additional SOAs were introduced (i.e., 0, ±40 and ±100 ms; see Figure 6B). A one-way repeated measures ANOVA revealed a significant main effect of SOA on the proportion of trials judged as “visual first” (*F*_(2.4,14.6)_ = 138.460, *p* < 0.001), and Bonferroni corrected *post hoc* analyses found that the performance during the 0 ms SOA was significantly different from both the −200 ms (auditory first) and +200 ms (visual first) SOA (*p* < 0.001; Figure 9B). Rats perceived the synchronous audiovisual stimuli to be “visual first” for nearly half of the trials (51.6 ± 4.4%; Figure 9B). When the auditory stimulus preceded or followed the visual stimulus by 100 ms, the rats were able to correctly judge the temporal order of the audiovisual stimuli on the majority of trials (−100 ms SOA: 74.3 ± 2.7%; +100 ms SOA: 74.1 ± 2.1%; Figure 9B).

**Figure 10:**
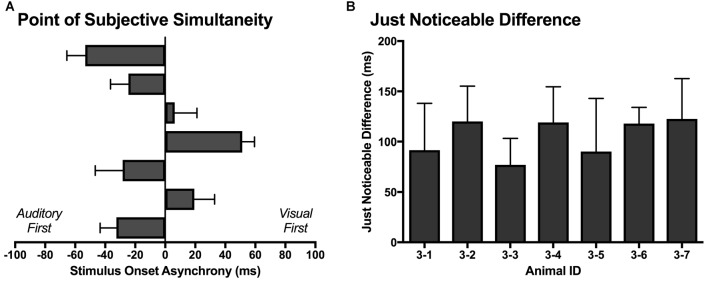
**The point of subjective simultaneity (PSS) and just noticeable difference (JND) derived from the temporal order judgment task. (A)** For each rat (*n* = 7; 3–1 to 3–7, plotted in ascending order), its PSS; i.e., the actual timing of the audiovisual stimuli when the observer is most unsure of the temporal order (Keetels and Vroomen, 2012) was determined. **(B)** For each rat (*n* = 7; 3–1 to 3–7), the metric of JND (i.e., the smallest interval between the separately presented auditory and visual stimuli that can be detected reliably) was calculated by taking the difference between the SOAs at which 25% and 75% of the responses were considered “visual-first” and then dividing by two (Vroomen and Stekelenburg, 2011). The PSS and JND were determined for each of the five testing days, and then averaged to provide a representative metric for each rat. Results are displayed as mean ± SEM.

Similar to temporal order judgment tasks performed by humans (Navarra et al., 2005; Vroomen and Stekelenburg, 2011; Keetels and Vroomen, 2012; Chen and Vroomen, 2013), the PSS and JND were calculated for each rat over its five testing days. As shown in Figure 10A, the PSS varied across rats, with values ranging from −53 ms (auditory first) to 51 ms (visual first). On average, the PSS was −8.8 ± 13.6 ms, which suggests that the rats tended to perceive the synchronously presented audiovisual stimuli as though the auditory stimulus was delivered slightly in advance of the visual stimulus. When averaged across all seven rats, the JND was 105 ± 7 ms, with values ranging from 77 ms to 122 ms (Figure 10B).

## Discussion

To our knowledge, the present study represents the first investigation into the development and implementation of behavioral paradigms to assess the perception of audiovisual temporal synchrony in rodents. Using operant conditioning, rats were trained to perform: (1) a simultaneity judgment task in which they reported whether audiovisual stimuli at various SOAs were presented at the same moment in time or at different times; and (2) a temporal order judgment task in which they reported whether they perceived the auditory or visual stimulus to be presented first. Rats were able to learn both tasks, and the resultant psychophysical curves were similar to those reported in humans (Zampini et al., 2005a; Vatakis et al., 2008b). In addition, we conducted the first investigation of how neurons in the rat multisensory cortex integrate audiovisual stimuli presented at different SOAs. By comparing the spiking activity in response to the audiovisual stimuli presented at the various SOAs, we confirmed that the profile of neuronal activity in the rat V2L cortex was similar to that recorded in various multisensory brain regions of different species. Overall, our collective findings suggest that the rat represents an effective model for studying audiovisual temporal synchrony at both the neuronal and perceptual level.

### Behavioral Assessments of Audiovisual Temporal Synchrony

A variety of experimental procedures have been developed to assess the ability of humans to determine the relative timing of combined auditory and visual stimuli presented at different SOAs by using a method of constant stimuli (Spence et al., 2001). The two procedures that have been used most often are the simultaneity judgment task and the temporal order judgment task. Although both of these tasks can assess an observer’s perception of the temporal synchrony of audiovisual stimuli, it is thought that these tasks reflect different underlying mechanisms (Vatakis et al., 2008b; Love et al., 2013) and may be subject to different kinds of response biases (Schneider and Bavelier, 2003; Vatakis and Spence, 2007; Vatakis et al., 2008b).

Typically, the simultaneity judgment task asks observers to judge whether audiovisual stimuli were presented at the same moment in time (i.e., synchronous) or at different moments in time (i.e., asynchronous), irrespective of whether the auditory or visual stimulus was presented first (Spence et al., 2001; Stone et al., 2001; Zampini et al., 2005a; Vatakis et al., 2008b; Stevenson et al., 2014; Binder, 2015). In contrast, although our simultaneity judgment task (Experiment 2) also required that rats judge whether the audiovisual stimuli were presented synchronously or asynchronously, we elected to have the visual stimulus always precede the auditory stimulus (and never vice-versa). This protocol choice was made because numerous studies on humans have shown that the PSS typically occurs when visual stimulus precedes the auditory stimulus by approximately 50 ms (Stone et al., 2001; Zampini et al., 2005a; Vatakis and Spence, 2008; Boenke et al., 2009; Vroomen and Stekelenburg, 2011; Stevenson et al., 2014). Although the experimental procedures differed between species, the performance results of the rats in the present study were similar to those of humans when compared to the “visual first” SOAs (Zampini et al., 2005a; Vatakis et al., 2008b; Stevenson et al., 2014). As predicted, rats were able to accurately (~80%) detect the difference between trials when audiovisual stimuli were presented synchronously vs. when the visual stimulus preceded the auditory by 200 ms (Figure 5B), and their performance scaled according to the interposed audiovisual SOAs. For example, similar to humans (Zampini et al., 2005a), the rats judged trials with a 10 ms SOA to be synchronous, whereas the majority (~70%) of trials at 100 ms SOA were perceived to be asynchronous (Figure 9A). Collectively, these results provide support for the face validity of our newly-developed simultaneity judgment task for rats. It is worth noting, however, that rats training on the simultaneity judgment task were susceptible to developing a response bias, which resulted in a longer-than-expected training duration. Interestingly, Vatakis and Spence (2007) described that response bias may manifest more when humans perform simultaneity judgment tasks compared to temporal order judgment tasks. Thus, in an effort to lessen the potential for response bias, and to evaluate the perception of temporal synchrony when an auditory stimulus was presented before- or after a visual stimulus, we developed a novel temporal order judgment task for rats.

In Experiment 3, the ability of rats to judge temporal order was assessed at SOAs of 0, ±40, ±100, ±200 ms, as these timing onsets not only matched those used in Experiment 2 but were similar to the SOAs used in testing human participants. Consistent with humans (Vatakis et al., 2008a,b), rats in the present study were able to accurately differentiate which modality was presented first when the SOAs were ±200 ms (Figure 6B). Moreover, when the timing difference between the stimuli was incrementally reduced, the rats showed a commensurate decline in performance toward chance levels (Figure 9B; see ~50% proportion of “visual first” responses when SOA was 0 ms). In addition to examining the psychophysical curve of response accuracy (Figure 9B), the PSS and JND were calculated from the temporal order judgment task. As shown in Figure 10A, the PSS values of the rats were variable, ranging from −53 ms (“auditory first”) to 51 ms (“visual first”); findings within the range of values reported in experiments conducted on humans (Zampini et al., 2003; Navarra et al., 2005; Vatakis and Spence, 2007; Vatakis et al., 2008b). Similar to Vatakis et al. (2008b), who found that the mean PSS value was 1 ms and −6 ms for synchronous and asynchronous speech monitoring, respectively, the mean PSS value for the rats was −8 ms (i.e., auditory was judged to precede visual). Moreover, the average JND value of 105 ± 7 ms indicates that rats were able to determine the temporal order of different sensory modalities similar to humans (Navarra et al., 2005; Vatakis et al., 2008a,b).

### Neural Basis of Audiovisual Temporal Processing?

Neuroimaging studies have provided insight into the brain regions activated during audiovisual processing tasks. For example, the insula, ventrolateral prefrontal cortex, and inferior parietal lobe (predominantly within the right hemisphere) have been shown to be engaged in the perception of audiovisual simultaneity (Bushara et al., 2001; Adhikari et al., 2013; Binder, 2015) and multisensory perception (Calvert and Thesen, 2004). Investigations into audiovisual temporal synchrony perception have found differences in the networks activated in response to synchronous and asynchronous stimuli. Consistent with temporal order judgment tasks in the visual domain (Davis et al., 2009), activation of both the left and right temporal parietal junction (TPJ) was observed, where the right temporal and parietal cortices, TPJ, as well as the right frontal and left parietal cortices showed greater activation to asynchronous perception in comparison to the synchronous perception of audiovisual stimuli (Adhikari et al., 2013). While differences in the degree of activation have been observed between synchronous and asynchronous perception, Binder (2015) demonstrated that simultaneity judgment tasks and temporal order judgment tasks activate similar cortical networks; however, the temporal order judgment task requires a greater amount of activation within the prefrontal, parietal lobules and occipito-temporal regions. This higher degree of neuronal activation is thought to be due to the additional cognitive operations that are required to judge which stimulus was presented first (Binder, 2015).

At this time, it is not possible to be certain which brain areas in the rat are responsible for audiovisual temporal synchrony perception, and whether these neuronal networks and patterns of activity differ during simultaneity- vs. temporal order judgment tasks. It is, however, reasonable to speculate that the V2L cortex may contribute to task performance. For example, as shown in the present study (Experiment 1), the rat V2L cortex—a well-established area responsive to audiovisual stimuli (Toldi et al., 1986; Barth et al., 1995; Wallace et al., 2004; Hirokawa et al., 2008; Xu et al., 2014; Schormans et al., 2016)—is sensitive to differences in the timing of combined audiovisual stimuli, such that spiking activity was greatest during trials when the visual stimulus preceded the auditory by 20–40 ms (Figures 7C, 8C). These results are fairly consistent with previous studies that recorded audiovisual-evoked spiking activity in the superior colliculus (cat (Meredith and Stein, 1986, 1996; Meredith et al., 1987; Perrault et al., 2005, 2012; Stanford et al., 2005) and guinea pig (King and Palmer, 1985)) as well as multisensory cortices (cat PLLS (Allman and Meredith, 2007; Allman et al., 2008b, 2009) and cat FAES (Meredith and Allman, 2009)), and further confirm that the timing of the stimuli play a critical role in the ability of the neurons to integrate the different sensory modalities (King and Palmer, 1985; Meredith and Stein, 1986; Perrault et al., 2005; Stanford et al., 2005; Miller et al., 2015). Although the V2L cortex has been shown to play an important role in audiovisual processing, future investigations are needed in order to assess audiovisual temporal processing at the single neuron level. As additional support of the potential role of the V2L cortex in the audiovisual temporal synchrony tasks, Hirokawa et al. (2008) demonstrated using local pharmacological inactivation that the V2L cortex was responsible for the improved reaction time to detect audiovisual stimulation (i.e., multisensory facilitation). That said, given the extra demands of decision-making in the audiovisual temporal synchrony tasks developed in the present study, it is likely that, in addition to the V2L cortex, areas of the prefrontal and posterior parietal cortices also influence perceptual judgments. Indeed, Raposo et al. (2014) demonstrated that the neurons in the posterior parietal cortex of rats dynamically-contributed to the performance of a multisensory decision-making task. Ultimately, our future studies will seek record the neural activity in the V2L cortex as rats perform the simultaneity- and temporal judgment tasks so as to further investigate the putative neural substrates contributing to the perception of audiovisual temporal synchrony.

## Author Contributions

ALS conducted all experiments; co-designed all experimental procedures; co-wrote the manuscript. KES assisted in data collection and design of behavioral procedures. AMQV and AT assisted in data collection and setup of behavioral procedures. MT assisted with the setup of the behavioral hardware and software; edited the manuscript. DS designed behavioral software; co-designed behavioral procedures; edited the manuscript. BLA co-designed experimental procedures; co-wrote the manuscript.

## Funding

This research support was provided by a Natural Sciences and Engineering Research Council (NSERC) Discovery grant and a Canadian Institutes of Health Research (CIHR) grant awarded to BLA.

## Conflict of Interest Statement

The authors declare that the research was conducted in the absence of any commercial or financial relationships that could be construed as a potential conflict of interest.

## Footnotes

^1^dstolz.github.io/epsych/
